# Outbreak of Influenza and Other Respiratory Viruses in Hospitalized Patients Alongside the SARS-CoV-2 Pandemic

**DOI:** 10.3389/fmicb.2022.902476

**Published:** 2022-06-13

**Authors:** Ilana S. Fratty, Shira Reznik-Balter, Ital Nemet, Nofar Atari, Limor Kliker, Hilda Sherbany, Nathan Keller, Michal Stein, Ella Mendelson, Michal Mandelboim

**Affiliations:** ^1^Central Virology Laboratory, Public Health Services, Ministry of Health and Sheba Medical Center, Ramat Gan, Israel; ^2^The Israel Center for Disease Control, Israel Ministry of Health, Ramat Gan, Israel; ^3^Sackler Faculty of Medicine, Department of Epidemiology and Preventive Medicine, Tel Aviv University, Tel Aviv, Israel; ^4^School of Health Sciences, Ariel University, Ariel, Israel; ^5^Pediatric Infectious Disease Unit, Sheba Medical Center, Ramat Gan, Israel

**Keywords:** respiratory viruses, influenza, outbreak, SARS-CoV-2, omicron variant

## Abstract

Influenza A and other respiratory viruses, circulate each winter and cause respiratory illness that can lead to severe complications in hospitalized patients. During the COVID-19 pandemic, only a few cases of respiratory viruses were detected in Israel. Our study applied RT-PCR to examine 13,674 samples collected from patients hospitalized with respiratory symptoms in 2019, 2020, and 2021 and the first half of the 2022 winter. A sharp increase in influenza A(H3N2) cases was observed in winter 2021-2022 as compared to 2020, followed by a sudden decrease in influenza cases after the detection of the SARS-CoV-2 omicron variant in Israel. Comparison of the area under the curve (AUC) of influenza infection rates during 7 consecutive winter seasons found that the minimal AUC between 2015 and 2020 was 281.1, while in 2021-2022, it was significantly lower (162.6 AUC; *p* = 0.0017), although the percentage of positive influenza cases was similar to those of previous years. The presented findings show how the dominance of influenza A(H3N2) abruptly ended upon circulation of the SARS-CoV-2 omicron variant. However, a post-COVID-19 influenza outbreak is possible, hence the planning of the next influenza vaccine is critical to ensure lower influenza-related hospitalization rates.

## Introduction

Each winter season, respiratory viruses, such as influenza, respiratory syncytial virus (RSV), and human metapneumovirus (hMPV), emerge and cause mild to severe symptoms such as fever, cough and other respiratory symptoms. The infection can lead to serious complications which can be lethal, especially in young children, pregnant women, and the elderly (Meningher et al., 2014; Glatman-Freedman et al., 2020; Kenmoe et al., 2020). These complications occur more frequently during winter seasons and pandemics (Klein et al., 2016).

COVID-19 has similar symptoms resembling influenza, including fever, headache, muscle ache and symptoms in the upper respiratory system such as coughing. Studies have shown that mortality rates in case of COVID-19 infection with the wild type SARS-CoV-2 may be even higher than infection with winter respiratory viruses (WHO, 2021; Hedberg et al., 2022). Overall, respiratory infection caused by either influenza, other respiratory viruses or COVID-19 can lead to serious complications such as cardiovascular events and bacterial co-infections that may be lethal (Piroth et al., 2021; Hedberg et al., 2022).

The emergence of COVID-19 in 2019-2020 was paralleled by a dramatic decrease in the prevalence of other respiratory viruses such as influenza, RSV and hMPV (Agca et al., 2021). Winter season 2020-2021 was considered historical, with the lowest rate of respiratory virus infection (Olsen et al., 2021). Although vaccination against SARS-CoV-2 led to a decrease in SARS-CoV-2 infections, still other respiratory viruses lack a compatible vaccine (Olsen et al., 2021; Khalil and Bernstein, 2022).

The Objective of the study was to examine the prevalence of influenza and other respiratory viruses among hospitalized patients before and during the SARS-CoV-2 pandemic, including during first, second and third anti-COVID-19 vaccination and novel variants penetration to Israel. This study also compared the rate of influenza infections among hospitalized patients in the winter seasons of 2015 to 2022 and found a particular phenomenon influenced by SARS-CoV-2 presence in Israel.

## Materials and Methods

### Patients and Samples

The samples included in this study were collected at Sheba Medical Centre (SMC), the largest tertiary medical center in Israel, which provides medical aid mostly to the population in the center of Israel. Demographic information of the patients (Supplementary Tables 1,2) shows similar distribution in gender and age between patients in the years examined.

The analysis between August 2019 and January 2022 included lab results of 13,674 nasopharyngeal samples collected from patients hospitalized at SMC due to respiratory illness and influenza-like symptoms. All samples were sent for routine clinical analysis to identify the presence of respiratory viruses.

For calculating the area under the curve (AUC), 25,448 nasopharyngeal samples were collected and analyzed. The data collected included the winter seasons of 2015-2016, 2016-2017, 2017-2018, 2018-2019, 2019-2020, 2020-2021, and 2021-2022.

### Area Under the Curve Calculations and Statistical Analysis

Calculation of the AUC of infection rates was conducted using a trapezoidal numerical integration. The statistical analysis between seasons was performed using the *T*-test. Both calculations were performed with MATLAB MathWorks, Inc. (United States).

### Viral RNA Extraction

Until January 2020, nucleic acids of viral RNA/DNA were extracted using MagNA PURE 96 (Roch, Manheim, Germany). After January 2020, viral RNA/DNA was extracted using the STARMag Viral DNA/RNA 200C universal kit (Seegene Inc., South Korea).

### Real Time PCR Assay

RNA/DNA of influenza A(H1N1pdm or H3N2)/B, RSV, HMPV, parainfluenza, and adenoviruses was detected using real-time reverse transcription-PCR (rRT-PCR) assay by Taqman chemistry, as previously described (Heim et al., 2003; Meningher et al., 2014; Pando et al., 2021). Shortly, the master mix was prepared with Ambion Ag-Path master mix (Life technologies, United States) in multiplex reactions. Two different multiplex reactions were performed: one that included primers for influenza A(H3N2), influenza A (H1N1pdm), influenza B, RSV and the other containing primers for HMPV, parainfluenza and for the DNA virus adenovirus. The RT-PCR assay was performed on an ABI 7500 instrument (Thermo Fisher Scientific, United Kingdom). From January 2020, rRT-PCR assays were also performed using the AllplexTM RV essential assay (Seegene Inc., South Korea) in a CFX Real-time PCR system (Bio-Rad, United States) (Folgueira et al., 2019).

## Results

### Influenza and SARS-CoV-2 in 2019-2022

**FIGURE 1:**
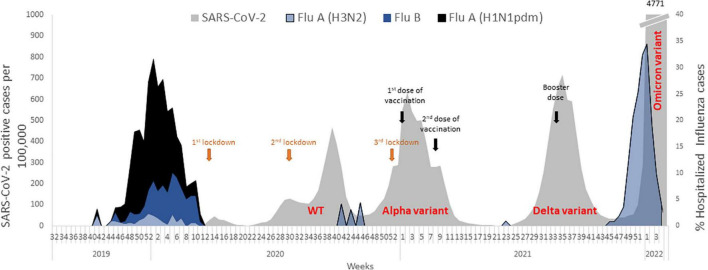
Incidence of influenza subtypes in winter 2019-2020, before and during the SARS-CoV-2 pandemic. After SARS-CoV-2 emerged in 2020, almost no influenza cases were found although first, second and third vaccination influenced the number of SARS-CoV-2 positive cases.

Until the emergence of SARS-CoV-2, the 2019-2020 winter was classified as a difficult influenza season with high infection rates, longer symptom durations and more hospitalizations compared to previous winter seasons (Xia et al., 2020; European Centre for Disease Prevention and Control [ECDC], 2022). The associated wave of hospitalized patients began in October 2019 and persisted for 18 weeks (Figure 1). In parallel, in December 2019, SARS-CoV-2 was first detected and rapidly spread worldwide, affecting the population in Israel as well (Figure 1). In 2020, almost no cases of influenza infection were observed worldwide while the prevalence of the SARS-CoV-2 alpha variant (B.1.1.7) increased toward the end of 2020 (European Centre for Disease Prevention and Control [ECDC], 2022; Friedman et al., 2022). After the vaccination operation in January (in Israel) 2021, SARS-CoV-2 circulation waned until the SARS-CoV-2 delta variant (B.1.617.2) emerging in July–August 2021. Later, in September another vaccination operation was initiated and SARS-CoV-2 incidence dropped again. By October 2021, only a few cases of SARS-CoV-2 were detected, while the number of positive cases of influenza in hospitalized patients increased. The number of influenza infections later declined gradually when the SARS-CoV-2 omicron variant (B.1.1.529) began to circulate in January 2022. From this point, the percentage of hospitalized influenza-positive patients dramatically dropped within 3 weeks only.

### Influenza Circulation Between 2015 and 2022

**FIGURE 2:**
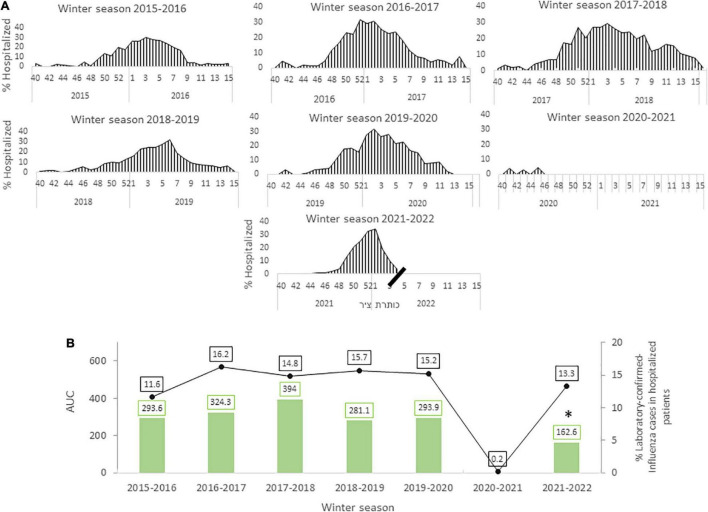
Circulation of influenza from week 40 to week 15 the following year for each winter season between 2015 and 2022. A comparison of the area under the curve (AUC) from winter season 2015 to winter season 2022 **(A)**. The percentage of influenza-positive cases among hospitalized patients in each winter season (green columns) alongside the total number of hospitalized patients tested for influenza in each year (black line). *p < 0.0017 **(B)**.

To compare the early part of the 2021-22 influenza winter season with previous winter seasons dating back until 2015-2016, we analyzed the positive influenza cases detected among hospitalized patients during each week of each winter season (Figure 2A). Then, we calculated the area under the curve (AUC) of infection rates using a trapezoidal numerical integration.

Only a few cases of influenza were detected by the end of January and February 2022 (data not shown). The AUC of winter season 2021-2022 influenza infection rates (so far) was significantly lower (*p* < 0.0017) than that of previous years even though the percentage of influenza hospitalized patients was similar to those of seasons up until 2019-2020 (Figure 2B).

### Incidence of Other Respiratory Viruses in Winter 2021-2022

**FIGURE 3:**
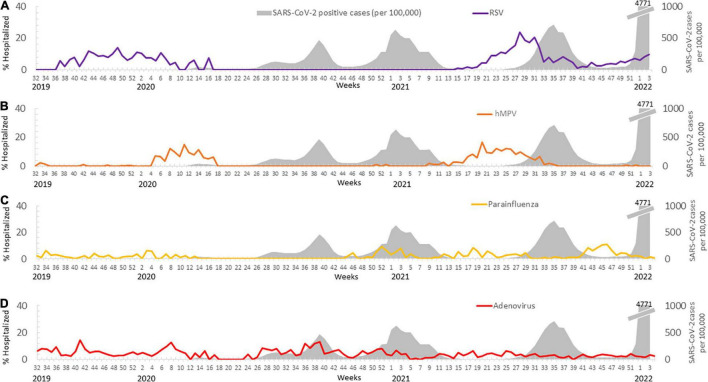
The rate of cases carrying respiratory viruses detected before and during the SARS-CoV-2 pandemic. The percentage of hospitalized patients carrying RSV **(A)**, parainfluenza **(B)**, HMPV **(C)**, and adenovirus **(D)** during the various waves of the SARS-CoV-2 pandemic (in gray).

The circulation of the main respiratory viruses each winter are presented in Figure 3. The gray area in Figures 3A–D indicates the SARS-CoV-2 waves, which began in Israel in February 2020. A rise in cases of RSV was mainly observed in the winter season of 2019-2020, however, when SARS-CoV-2 began to circulate in February 2020, RSV cases vanished almost completely (Figure 3A). RSV cases rose again after the decline in SARS-CoV-2 infection rates in May 2021, but then declined at the onset of the third wave of SARS-CoV-2 in August 2021. In the same period hMPV showed similar patterns to RSV and was also influenced by the presence of SARS-CoV-2 (Figure 3B). Parainfluenza cases increased in the 2020-2021 winter season (Figure 3C), but the overall number of positive cases in 2020 was relatively low compared to 2021 (Supplementary Table 3). Incidence of parainfluenza decreased in July 2021, and then rose in October 2021 when the winter season began (Figure 3C).

The prevalence of adenovirus remained constant over the years 2019-2022, regardless of SARS-CoV-2 infection rates (Figure 3D).

## Discussion

In December 2019, SARS-CoV-2 emerged, spread rapidly and was first detected in Israel in February 2020. Safety measurements, such as wearing protective masks, social distancing and lockdowns, were implemented worldwide, which all contributed to a reduction in the number of SARS-CoV-2 cases globally, as well as in Israel (Last, 2020; Mattia et al., 2021). In parallel to the emergence of COVID-19, the rate of infections by respiratory viruses decreased dramatically (Olsen et al., 2021) after a difficult winter season with influenza A(H1N1pdm), followed by influenza B and sporadic incidents of influenza A(H3N2) (Figure 1).

This study focused on hospitalized patients; hence, it was limited to severe cases. However, a report of the Israel Center for Disease Control showed similar influenza infection patterns in its sentinel surveillance in Israel (Israel Center for Disease Control [ICDC], 2022). According to a WHO update, North America and Europe experienced a rapid increase in influenza cases between November and December 2021, followed by a decrease in January 2022 (World Health Oraganization [WHO], 2022). Interestingly, in Europe the number of positive influenza cases was lower in the winter season of 2021-2022 compared to previous years (European Centre for Disease Prevention and Control [ECDC], 2022).

In Israel, the first COVID-19 lockdown was implemented in Israel in March 2020. Subsequently, the number of cases of COVID-19 waned then rose again. By September 2020 a second lockdown was implemented, which was followed by a decrease in COVID-19 cases. Later that year, the presence of the alpha variant led to a third lockdown in December 2020. In this period preceding the lockdown (March 2020 to December 2020) only sporadic cases of respiratory viruses, such as influenza, RSV, parainfluenza and HMPV, were detected in hospitalized patients (Figures 1, 3A–C).

When the vaccination operation began in December 2020-January 2021, the incidence of COVID-19 waned almost completely and restrictions were lifted. Children returned to frontal learning and social distancing ended. During this period, still no cases of influenza were detected, probably because it was the end of the winter season. In contrast to influenza, the number of RSV cases rose off-season in May 2021 and did not decline until August 2021. Similarly, hMPV cases increased after a year with only sporadic reports of infection. Parainfluenza infection rates were mostly low when COVID-19 rose; while fluctuations in infection rates were noted in the winter season of 2020-2021, they remained significantly low in 2020 compared to 2021 (Supplementary Table 3). A comparison between 2019, 2020, and 2021 (2022 was not included since it is still ongoing) showed significantly fewer cases of hMPV and parainfluenza in 2020 (Supplementary Table 3). These findings align with other studies that showed significantly lower incidences of respiratory viruses during the pandemic (Mattia et al., 2021; Olsen et al., 2021). However, infection rates of RSV and adenovirus were similar in 2019, 2020 and 2021. The prevalence of adenovirus in hospitalized patients (Figure 3D) was not influenced by SARS-CoV-2 waves and remained similar to those seen before the COVID-19 pandemic (Olsen et al., 2021). It is important to note that the positive cases of RSV detected in 2020 were part of the 2019-2020 winter season before the pandemic. Later in June 2021, off-season RSV and hMPV infections in hospitalized patients were documented (Figure 3A) as reported by others (Olsen et al., 2021; Weinberger Opek et al., 2021; Friedman et al., 2022). These increases in RSV and hMPV infections are presumably due to lack of previous exposure to RSV in the population (Olsen et al., 2021; Weinberger Opek et al., 2021).

In August 2021, the third vaccination operation began, which led to a decrease in SARS-CoV-2 cases, which was followed by detection of the first cases of influenza in hospitalized patients, with numbers growing and peaking in December 2021. Our comparison in Figure 2B shows a similar percentage of hospitalized patients between 2015 and 2019 winter seasons, despite the fact that the analyzed 2021-2022 period was shorter than the full seasons studied from previous years. In other words, the winter season of 2021-2022 was characterized with a high percentage of positive influenza cases in a very short period of time.

Herein, we suggest that the immediate drop in influenza cases during circulation of SARS-CoV-2 variant omicron indicates an antagonistic effect on influenza A(H3N2) by SARS-CoV-2. This antagonistic effect was shown in previous studies with other respiratory viruses such as RSV and influenza (Ayegbusi et al., 2019; Drori et al., 2020; Kaaijk et al., 2022), and may act as a form of resistance against a second virus when the host is infected by the first virus. Viral interference was suggested to play a part in reduction of RSV and/or influenza (Drori et al., 2020; Kaaijk et al., 2022). Rhinovirus, for instance, was observed less frequently than expected in the presence of influenza and was also shown in Norway and France to inhibit the circulation of influenza A(H1N1pdm) (Casalegno et al., 2010; Anestad and Nordbo, 2011). Furthermore, as seen in previous studies, the immune system can mediate upregulation of anti-viral proteins to provide immunity against other respiratory viruses (Ayegbusi et al., 2019; Drori et al., 2020). Additionally, severity of the disease was shown to decrease with RSV-influenza co-infections compared to single infection (Ayegbusi et al., 2019). In fact, most of the complications of respiratory virus infection in the lung arise from bacterial co-infections and not viral co-infections (Manna et al., 2022).

The SARS-CoV-2 pandemic and its inhibitory effect on influenza infection can also be compared to the 2009 influenza A(H1N1pdm), which was accompanied by a marked drop in infection rates of other respiratory viruses. More specifically, the 2009 influenza A(H1N1pdm) brought to a 2.5-month delay in the onset of the RSV season. It also led to a decrease in HMPV infections (Meningher et al., 2014), similar to the effect of SARS-CoV-2.

These conclusions do not contrast those of Zhang et al., who showed coinfection with SARS-CoV-2 and influenza A(H1N1pdm) but did not examine influenza A(H3N2) (Zhang et al., 2021). In our analysis of 2021-22 samples, we identified only influenza A(H3N2) cases and no influenza A(H1N1pdm) incidences. It has been suggested that the influenza clades circulating in winter season 2019-2020, including influenza A(H1N1pdm), will return. However, the sporadic cases of influenza A(H3N2) have been observed are still circulating now in 2021-2022 (Laurie and Rockman, 2021). Importantly, influenza A(H3N2), the influenza strain circulating in Israel, was part of the quadrivalent influenza vaccine given to the population in Israel. Yet, to date (05/02/2022), only 723,192 individuals, mostly older than 65-year-old, were vaccinated in Israel (∼0.08% of the population in Israel) (Israel Ministry of Health [IMoH], 2021). Multiple methods for developing an influenza vaccine are still under investigation, but, generally, these vaccines are less customized for influenza A(H3N2) (Khalil and Bernstein, 2022). Hence, the planning of the next anti-influenza vaccine will be great significance for hospitalized patients with respiratory symptoms caused by influenza (Kalil and Thomas, 2019).

To conclude, circulation of influenza among hospitalized patients began in Israel in September-October 2021 and rapidly became rampant, compensating for the lost year of 2020 when almost no influenza cases were identified. However, the high number of patients hospitalized with influenza declined very quickly with the penetration of the SARS-CoV-2 omicron variant, suggesting an antagonistic affect. In parallel, off-season presence of RSV, parainfluenza and hMPV were affected by the COVID-19 pandemic, as opposed to adenovirus, which did not show significant difference between 2019 and 2022. These findings are important for forecasting future influenza seasons and for the preparation of a compatible vaccine for the coming influenza season, with or without new SARS-CoV-2 variants circulation.

## Data Availability Statement

The raw data supporting the conclusions of this article will be made available by the authors, without undue reservation.

## Author Contributions

IF: study design and data analysis. SR-B: statistical analysis. IF and MM: data interpretation. IF, IN, NA, LK, and HS: conducted the experiments. MM, MS, EM, and NK: critical revision of manuscript. All authors have read and agreed to the published the version of the manuscript.

## Conflict of Interest

The authors declare that the research was conducted in the absence of any commercial or financial relationships that could be construed as a potential conflict of interest.

## Publisher’s Note

All claims expressed in this article are solely those of the authors and do not necessarily represent those of their affiliated organizations, or those of the publisher, the editors and the reviewers. Any product that may be evaluated in this article, or claim that may be made by its manufacturer, is not guaranteed or endorsed by the publisher.

## References

[B1] Agca H. Akalin H. Saglik I. Hacimustafaoglu M. Celebi S. Ener B. (2021). Changing epidemiology of influenza and other respiratory viruses in the first year of COVID-19 pandemic. *J. Infect. Public Health* 14 1186–1190. 10.1016/j.jiph.2021.08.004 34399190

[B2] Anestad G. Nordbo S. A. (2011). Virus interference. Did rhinoviruses activity hamper the progress of the 2009 influenza A (H1N1) pandemic in Norway? *Med. Hypotheses* 77 1132–1134. 10.1016/j.mehy.2011.09.021 21975051

[B3] Ayegbusi O. T. Ajagbe O. A. Afowowe T. O. Aransi A. T. Olusola B. A. Awogbindin I. O. (2019). Virus genes and host correlates of pathology are markedly reduced during respiratory syncytial and influenza virus co-infection in BALB/c mice. *Heliyon* 5:e01094. 10.1016/j.heliyon.2018.e01094 30623128 PMC6319304

[B4] Casalegno J. S. Ottmann M. Duchamp M. B. Escuret V. Billaud G. Frobert E. (2010). Rhinoviruses delayed the circulation of the pandemic influenza A (H1N1) 2009 virus in France. *Clin. Microbiol. Infect.* 16 326–329. 10.1111/j.1469-0691.2010.03167.x 20121829

[B5] Drori Y. Jacob-Hirsch J. Pando R. Glatman-Freedman A. Friedman N. Mendelson E. (2020). Influenza a virus inhibits RSV infection *via* a two-wave expression of IFIT proteins. *Viruses* 12:1171. 10.3390/v12101171 33081322 PMC7589235

[B6] European Centre for Disease Prevention and Control [ECDC] (2022). *Flu News Europe.* Geneva: World health Organization.

[B7] Folgueira L. Moral N. Pascual C. Delgado R. (2019). Comparison of the Panther Fusion and Allplex assays for the detection of respiratory viruses in clinical samples. *PLoS One* 14:e0226403. 10.1371/journal.pone.0226403 31881030 PMC6934309

[B8] Friedman N. Levy N. Kaplan O. Padeh G. Krupik K. Jacob R. (2022). Pediatric hospitalizations after school reopening during the SARS-CoV-2 Alpha (B.1.1.7) variant spread: a multicenter cross-sectional study in Israel. *Clin. Infect. Dis.* 10.1093/cid/ciac065 [Online ahead of print] 35092684 PMC8807310

[B9] Glatman-Freedman A. Kaufman Z. Applbaum Y. Dichtiar R. Steiman A. Gordon E. S. (2020). Respiratory Syncytial Virus hospitalization burden: a nation-wide population-based analysis, 2000-2017. *J. Infect.* 81 297–303. 10.1016/j.jinf.2020.05.078 32504738

[B10] Hedberg P. Karlsson Valik J. Van Der Werff S. Tanushi H. Mendez A. R. Granath F. (2022). Clinical phenotypes and outcomes of SARS-CoV-2, influenza, RSV and seven other respiratory viruses: a retrospective study using complete hospital data. *Thorax* 77 154–163. 10.1136/thoraxjnl-2021-216949 34226206 PMC8260304

[B11] Heim A. Ebnet C. Harste G. Pring-Akerblom P. (2003). Rapid and quantitative detection of human adenovirus DNA by real-time PCR. *J. Med. Virol.* 70 228–239. 10.1002/jmv.10382 12696109

[B12] Israel Center for Disease Control [ICDC] (2022). Available online at: https://www.health.gov.il/flu_weekly/flu_14032020e.pdf (accessed on April 2, 2022).

[B13] Israel Ministry of Health [IMoH] (2021). *Corona Virus in Israel – General Situation.* Available online at: Https://datadashboard.health.gov.il/COVID-19/ [accessed on May 25, 2022].

[B14] Kaaijk P. Swaans N. Nicolaie A. M. Bruin J. P. Boxtel R. A. Lange M. (2022). Contribution of influenza viruses, other respiratory viruses and viral co-infections to influenza-like illness in older adults. *Viruses* 14:797. 10.3390/v14040797 PMC9024706 35458527

[B15] Kalil A. C. Thomas P. G. (2019). Influenza virus-related critical illness: pathophysiology and epidemiology. *Crit. Care* 23:258. 10.1186/s13054-019-2539-x 31324202 PMC6642581

[B16] Kenmoe S. Kengne-Nde C. Ebogo-Belobo J. T. Mbaga D. S. Fatawou Modiyinji A. Njouom R. (2020). Systematic review and meta-analysis of the prevalence of common respiratory viruses in children < 2 years with bronchiolitis in the pre-COVID-19 pandemic era. *PLoS One* 15:e0242302. 10.1371/journal.pone.0242302 33180855 PMC7660462

[B17] Khalil N. Bernstein D. I. (2022). Influenza vaccines: where we are, where we are going. *Curr. Opin. Pediatr.* 34 119–125. 10.1097/MOP.0000000000001103 35034078

[B18] Klein E. Y. Monteforte B. Gupta A. Jiang W. May L. Hsieh Y. H. (2016). The frequency of influenza and bacterial coinfection: a systematic review and meta-analysis. *Influenza Other Respir. Viruses* 10 394–403. 10.1111/irv.12398 27232677 PMC4947938

[B19] Last M. (2020). The first wave of COVID-19 in Israel-Initial analysis of publicly available data. *PLoS One* 15:e0240393. 10.1371/journal.pone.0240393 33119605 PMC7595440

[B20] Laurie K. L. Rockman S. (2021). Which influenza viruses will emerge following the SARS-CoV-2 pandemic? *Influenza Other Respir. Viruses* 15 573–576. 10.1111/irv.12866 33955176 PMC8242426

[B21] Manna S. McAuley J. Jacobson J. Nguyen C. D. Ullah M. A. Sebina I. (2022). Synergism and antagonism of bacterial-viral coinfection in the upper respiratory tract. *mSphere* 7:e009841. 10.1128/msphere.00984-21 35044807 PMC8769199

[B22] Mattia G. D. Nenna R. Mancino E. Rizzo V. Pierangeli A. Villani A. (2021). During the COVID-19 pandemic where has respiratory syncytial virus gone? *Pediatr. Pulmonol.* 56 3106–3109. 10.1002/ppul.25582 34273135 PMC8441855

[B23] Meningher T. Hindiyeh M. Regev L. Sherbany H. Mendelson E. Mandelboim M. (2014). Relationships between A(H1N1)pdm09 influenza infection and infections with other respiratory viruses. *Influenza Other Respir. Viruses* 8 422–430. 10.1111/irv.12249 24698156 PMC4181801

[B24] Olsen S. J. Winn A. K. Budd A. C. Prill M. M. Steel J. Midgley C. M. (2021). Changes in influenza and other respiratory virus activity during the COVID-19 pandemic-United States, 2020-2021. *Am. J. Transplant* 21 3481–3486. 34624182 10.1111/ajt.16049 PMC8653380

[B25] Pando R. Stern S. Nemet I. Glatman-Freedman A. Sefty H. Zuckerman N. S. (2021). Diversity in the circulation of influenza A(H3N2) viruses in the northern hemisphere in the 2018-19 season. *Vaccines* 9:375. 10.3390/vaccines9040375 33924296 PMC8069444

[B26] Piroth L. Cottenet J. Mariet A. S. Bonniaud P. Blot M. Tubert-Bitter P. (2021). Comparison of the characteristics, morbidity, and mortality of COVID-19 and seasonal influenza: a nationwide, population-based retrospective cohort study. *Lancet Respir. Med.* 9 251–259. 10.1016/S2213-2600(20)30527-0 33341155 PMC7832247

[B27] Weinberger Opek M. Yeshayahu Y. Glatman-Freedman A. Kaufman Z. Sorek N. Brosh-Nissimov T. (2021). Delayed respiratory syncytial virus epidemic in children after relaxation of COVID-19 physical distancing measures, Ashdod, Israel, 2021. *Euro Surveill.* 26:2100706. 10.2807/1560-7917.ES.2021.26.29.2100706 34296678 PMC8299746

[B28] WHO (2021). Available online at: https://www.who.int/mongolia/multi-media/item/common-symptoms-of-covid-19 (accessed October 27, 2021).

[B29] World Health Oraganization [WHO] (2022). *Influenza Update Number 417.* Available online at: https://www.who.int/teams/global-influenza-programme/surveillance-and-monitoring/influenza-updates/current-influenza-update [accessed on May 16, 2022].

[B30] Xia Z. Yang L. Li N. Nie B. Wang H. Xu H. (2020). Seasonal influenza activity in young children before the COVID-19 outbreak in Wuhan, China. *Transbound. Emerg. Dis.* 67 2277–2279. 10.1111/tbed.13799 32881325 PMC7461219

[B31] Zhang A. J. Lee A. C. Chan F. W. Liu F. Li C. Chen Y. (2021). Coinfection by severe acute respiratory syndrome coronavirus 2 and influenza A(H1N1)pdm09 virus enhances the severity of pneumonia in golden syrian hamsters. *Clin. Infect. Dis.* 72 e978–e992. 10.1093/cid/ciaa1747 33216851 PMC7717201

