# Supplementary material for: Outbreak of Influenza and Other Respiratory Viruses in Hospitalized Patients Alongside the SARS-CoV-2 Pandemic
Source: Front Microbiol. 2022 Jun 13;13:902476. doi: 10.3389/fmicb.2022.902476 (PMC9235518; doi:10.3389/fmicb.2022.902476)
Supplement: Supplementary file 1 [file Table_1.DOCX]

**Supplementary materials**

**Supplementary Table S1**. Age distribution of positive hospitalized patients with influenza between August 2019 to January 2022

| **Age/Year** | **2019** | **2020** | **2021** | **Jan-2022** |
| --- | --- | --- | --- | --- |
| **0-2** | 395 | 573 | 852 | 113 |
| **3-9** | 101 | 251 | 252 | 52 |
| **10-14** | 38 | 89 | 93 | 14 |
| **15-24** | 100 | 197 | 243 | 50 |
| **25-44** | 283 | 458 | 476 | 102 |
| **45-64** | 427 | 691 | 947 | 166 |
| **65≤** | 1164 | 1966 | 2938 | 590 |

**Supplementary Table S2**. Gender distribution of positive hospitalized patients with influenza between August 2019 to January 2022

| **Gender/Year** | **2019** | **2020** | **2021** | **Jan-2022** |
| --- | --- | --- | --- | --- |
| **Male** | 1351 | 2155 | 3155 | 573 |
| **Female** | 1155 | 2079 | 2658 | 517 |

**Supplementary Table S3.** Comparison of the presence of respiratory viruses in hospitalized patients in 2019,2020, 2021 and early 2022

| **Jan-22** | **2021** | **2020** | **2019** |  |
| --- | --- | --- | --- | --- |
| 0 | 206 | 63* | 297 | **hMPV** |
| 0 | 193 | 38* | 191 | **Parainfluenza** |
| 35 | 334 | 219 | 494 | **RSV** |
| 11 | 277 | 152 | 433 | **Adenovirus** |

**p*≤0.05
